# Gall Stones

**Published:** 1875-12

**Authors:** 


Gall-Stones—Their Removal by a Singular Method.—M. Paulet (*Allg. Wiener Med. Zeitung, The Clinic*, September 4, 1875) reports the following:

A woman, aged forty two, a mother of eight children, began in November, 1874, to feel a severe pain in the right side. On examination there was found a somewhat diffuse tumor about the size of a child's head, which extended from the umbilicus to the anterior superior spinous process of the ilium. As the case was attended by fever, it was diagnosed as one of suppurative ovaritis. In December it was determined to open the tumor with Vienna paste. In three days the skin was destroyed, and in seven the aponeuroses. An exploratory incision was now made, from which a little fluid escaped, but the needle came in contact with numerous hard bodies, which on removal proved to be gall-stones. Forty of these were then taken out, and a large one found almost encapsulated. It was broken down and extracted without much difficulty, when some bile was also discharged. This biliary fistula soon closed, and in March, 1875, the patient had entirely recovered.—*The Physician and Pharmacist.*

